# Mechanical Behavior of Foam-Filled Bamboo Composite Tubes under Axial Compression

**DOI:** 10.3390/polym14102006

**Published:** 2022-05-13

**Authors:** Yang Wei, Shuaifeng Tang, Si Chen, Qiudong Wang, Jiaqing Wang

**Affiliations:** 1 College of Civil Engineering, Nanjing Forestry University, Nanjing 210037, China; tsf941028@163.com (S.T.); cs0714@njfu.edu.cn (S.C.); qdwang@njfu.edu.cn (Q.W.); jiaqingw@njfu.edu.cn (J.W.); 2 Baosteel Engineering & Technology Group Co., Ltd., Shanghai 201999, China

**Keywords:** foam-filled, bamboo composite tube, axial compression, energy absorption

## Abstract

In this paper, a new type of polyurethane foam-filled bamboo composite tube is proposed. Axial compression tests were carried out on unfilled and polyurethane foam-filled bamboo composite tubes. The effects of the foam filler, diameter (50 and 100 mm) and number of winding layers (10, 15 and 20 layers) on the failure mode and energy absorption capacity of the tubes were studied. The test results showed that the failure mode of the unfilled tube was buckling failure, while that of the foam-filled tube was pressure-bearing failure, and the latter was more abrupt. The foam filler enhanced the stability of the wall of the unfilled tube. The interaction between them further increased the bearing capacity of the foam-filled tube and showed a higher platform load at a later stage. In terms of the absorbed energy, specific absorbed energy and average crush load, not all foam-filled tubes were superior to unfilled tubes. However, reducing the height of the bamboo composite tube and increasing the number of winding layers of the bamboo composite tube can effectively increase the positive effect of the foam filler on energy absorption.

## 1. Introduction

With the frequent occurrence of various accidents, structural crashworthiness has become a major concern in many fields. Therefore, the energy absorber plays an important role in protecting important structures. Foam material has the advantages of cost-effectiveness, light weight and large deformation capacity. Moreover, some studies have put forward that injecting rigid polyurethane into the soil can achieve the effect of seismic risk mitigation [1,2,3]. Combined with thin-walled tubes, it can form a very good energy-absorbing structure. This structure is mainly composed of high-performance steel, alloy and aluminum foam. At present, most of the research is focused on foam-filled metal tube structures [4,5,6,7]. Duarte et al. [8] used polymer aluminum alloy foam as a substitute for traditional closed-cell aluminum foam as aluminum alloy thin-walled square tube packing. The results showed that the foam packing could stabilize the wall of the tube and prevent the thin-walled tube from developing the unstable integral bending mode or mixed buckling mode. The composite tubes finally showed progressive folding deformation. Li et al. [9] studied five types of aluminum foam-filled tubes, including foam-filled single circular and square tubes, foam-filled double circular and square tubes and corner foam-filled square tubes. Their load–deformation characteristics and energy absorption capacities were compared. The foam-filled aluminum alloy tube structure had a higher compression efficiency and energy absorption efficiency and was suitable for collision-resistant structures.

Recently, environmental protection has become a hot issue and one of the main factors to be considered in engineering design, and thus, researchers have been considering the application of natural fiber-based composites. Natural fibers include silk, flax, hemp, etc. [10,11]. Natural fiber-based composites have the advantages of low cost, low density, recyclability and biodegradability. To date, a few studies have focused on unfilled and foam-filled natural fiber tubes. Eshkoor et al. [12,13] and Oshkovr et al. [14] reported the failure mode and failure characteristics of silk epoxy composite tubes. Buckling and the formation of plastic hinges were the two main characteristics of natural silk epoxy tubes, which eventually showed catastrophic failure. Yan et al. [15,16] studied the effects of the tube diameter, wall thickness, length-to-diameter ratio, triggering and foam filler on the axial crushing of natural flax epoxy composite tubes. The results showed that the flax epoxy resin composite tube was broken in a brittle manner, which was different from the catastrophic failure of the natural silk epoxy resin tube. The comprehensive performance of flax epoxy composite tubes as energy absorption devices is obviously better than that of natural silk epoxy composite tubes, and their specific energy absorption value is similar to that of aluminum alloy tubes; thus, their industrial application prospects are broad. The consumption of natural fibers as green materials instead of metal resources is of great significance for the sustainable development of civil engineering and ecological environments [17,18,19,20]. In addition, the energy absorption characteristics of foam-filled composite tubes [21] and natural bamboo tubes and a theoretical study of the crushing properties of foam-filled metal tubes [22] and natural fiber tubes [23] in transverse compression are presented. Due to the existence of foam filler, the foam-filled tube exhibits a gradual stable crushing mode, restraining the buckling deformation and delamination of the unfilled tube and ultimately improving the energy absorption capacity of the foam-filled tube during the flattening process.

Bamboo is considered a new material with great market potential among natural fibers [24,25,26,27] and has been widely used in civil engineering structures, such as short-span footbridges, low-rise houses, long-span roofs and construction envelopes [28,29,30,31]. The original bamboo tube, as a natural fiber tube structure, has attracted the attention of scholars [32,33,34]. Umer et al. [35] investigated the energy-absorbing characteristics of a range of lightweight bamboo-reinforced foam structures. The specific energy absorption of the bamboo tubes decreases with increasing tube length. Zou et al. [36] studied the energy absorption of moso bamboo through the drop weight test. The nodes and moisture content are the key factors affecting the energy absorption of bamboo, and the bamboo tube is a tubular structure with excellent mechanical properties and energy consumption. In addition, the combination of bamboo with other materials can achieve better performance [37,38,39,40,41,42,43]. In recent years, there has been some research on bamboo fiber tube confined concrete [44,45,46], but none of these studies involved foam-filled bamboo composite tubes.

In this paper, combining the excellent performance of bamboo and the good energy absorption of foam-filled tubes, a new type of polyurethane foam-filled bamboo composite tube is proposed. Unfilled bamboo composite tubes are prone to buckle, and the smaller the diameter is, the larger the slenderness ratio is, and the more prone they are to buckle [47]. Foam filler can improve the stability of thin-walled tube structures, but the foam is not suitable for single bearing, and the deformation recovery rate is high. Polyurethane foam-filled bamboo composite tubes can solve the defects of these two types of materials alone. Bamboo composite tubes can be used in building structures, such as column members of low-rise buildings, pressure members of truss structures and so on. Axial compression tests were carried out on polyurethane foam-filled bamboo composite tubes. The influence of the tube diameter, the number of winding layers and polyurethane foam filler on the failure modes and mechanical properties of polyurethane foam filler was studied. In addition, the effect of polyurethane foam on energy absorption characteristics was analyzed.

## 2. Materials

### 2.1. Bamboo Slices

Bamboo slices were prepared by slicing softened moso bamboo stem along the bamboo fiber, and the nominal thickness of each bamboo slice was 0.5 mm. A bamboo slice tensile test was carried out on five tensile specimens with displacement control, and the speed was 1.0 mm/min. The bamboo slice thickness was 0.5 mm, and the section size was 230 mm × 20 mm. Before fracture, the stress–strain curve of bamboo slices showed a linear change trend, and the failure mode was tooth failure with irregular cracks. The ultimate tensile strength, ultimate strain, elastic modulus and Poisson’s ratio were 95.03 MPa, 0.0077, 12.12 GPa and 0.29, respectively. The mechanical properties of bamboo slices are shown in Table 1.

The compression properties of bamboo slices were determined by three groups of glued compression test blocks with different wall thicknesses, with 10 specimens in each group. The compression test block was cut from three types of bamboo-wound composite tubes with three wall thicknesses. The cross-section size of the glued compression test block was 20 mm × 20 mm, and the wall thickness was the same as that of the bamboo composite tube. The test was carried out with constant-speed displacement control, and the loading speed was 0.3 mm/min. The typical failure modes of glued compression specimens can be divided into two types: buckling failure and splitting failure. The bearing capacity of the specimens with splitting failure was significantly lower than that of the same group of buckling failure specimens, and the change in wall thickness had a significant effect on the failure mode of small compressive specimens. With increasing wall thickness, the number of small compressive specimens with splitting failure increased significantly. The average ultimate compressive strengths of the specimens with wall thicknesses of 5 mm, 7.5 mm and 10 mm were 55.40 MPa, 59.73 MPa and 54.29 MPa, respectively.

### 2.2. Polyurethane Foam

**Figure 1:**
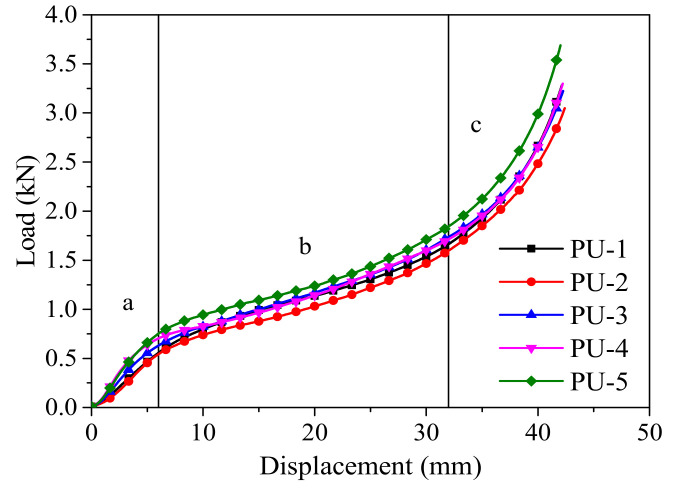
Load–displacement curves of polyurethane foam under static compression. (**a**) elastic stage; (**b**) platform stage; (**c**) dense stage.

The polyurethane foam used in this experiment is rigid polyurethane foam. According to the provisions of Chinese standard GB/T 8813-2020, five prismatic standard material samples of 100 mm × 100 mm × 50 mm were prepared. A static compressive test was carried out on a CMT4204 microcomputer-controlled electronic universal testing machine. The loading mode was controlled by constant velocity displacement, the loading speed was 2 mm/min, and the load was continuously loaded until the displacement reached 85% of the initial thickness of the specimen, that is, 42.5 mm. The polyurethane foam has good deformation recovery ability. The results of the polyurethane foam compression test are shown in Table 2. The theoretical density of polyurethane foam is 30.0–40.0 kg/m^3^, and the measured average density is 31.5 kg/m^3^. The compressive load–displacement curves of polyurethane foam are shown in Figure 1. Under uniaxial compression, polyurethane foam showed typical elastoplastic characteristics. The static load–displacement curves of polyurethane foam can be divided into three stages, i.e., elastic stage, platform stage and dense stage. As shown in Figure 1, when polyurethane foam was tested under axial compression, elastic deformation first occurred (stage a). When the elastic limit was exceeded, plastic deformation occurred in the platform stage (stage b), in which the load increased slowly with the increase in displacement, and this stage is plastic deformation. After the unloading test, the polyurethane foam could still be restored to 70% of the initial thickness, which is a typical elastoplastic deformation characteristic.

## 3. Experimental Program

### 3.1. New Composite Tube

**Figure 2:**
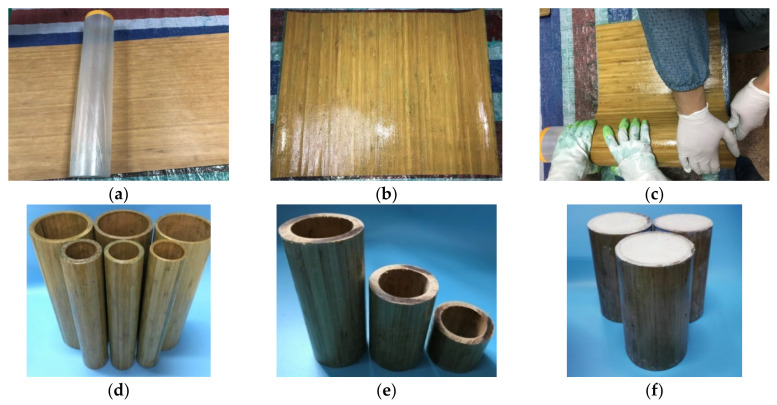
Manufacturing process of specimens. (**a**) Preparation of raw materials; (**b**) soakage of epoxy resin; (**c**) winding of bamboo slice; (**d**) demolding and curing; (**e**) cutting and polishing; (**f**) foam filling.

In order to overcome the shortcomings of the original bamboo tube, such as irregular size, limited diameter and large discrete performance, a composite bamboo tube made of hand-rolled bamboo slices is proposed in this paper. In addition, considering the good energy absorption of foam, polyurethane foam was used to fill the composite bamboo tube. The composite bamboo tube is prone to buckling when it is loaded alone. Filling it with foam can improve the structural stability of the thin-walled tube, but foam material is not suitable for loading alone, and the deformation recovery rate is high, so the two have good complementarity in performance. As shown in Figure 2, the manufacturing process of the polyurethane foam-filled bamboo composite tube is as follows:

(1) Preparation of raw materials: The finished side of pressed carbonized unidirectional bamboo slices, epoxy resin and several acrylic tube molds are the raw materials. The size of the bamboo slice is 360 mm × 450 mm, and the circular bamboo fiber tube is a continuous bamboo slice connected end to end. The surface of the bamboo slice is cleaned before production, and a layer of plastic film is wrapped around the acrylic tube mold to facilitate demolding in a later stage.
(2) Soakage of epoxy resin: The bamboo slices are laid flat on the working surface, both sides of the bamboo slices are soaked with the epoxy resin, and the operation is repeated until the epoxy resin saturates the bamboo slices.
(3) Winding of bamboo slices: The bamboo slices are tightly wrapped around the acrylic tube mold to ensure that the direction of bamboo fiber is parallel to the axis of the acrylic tube. There is no lap connection between two bamboo slices, and they are wound in turn and repeatedly squeezed to eliminate the interlayer gap. After wrapping the bamboo slices according to the number of layers, several layers of protective film are wrapped around them to prevent the bamboo slices from curling.
(4) Demolding and curing: The wrapped bamboo composite tube is placed at room temperature and left until the epoxy resin is completely hardened and separated from the acrylic tube. It is then placed at room temperature for curing.
(5) Cutting and polishing: The bamboo composite tube is cut according to the design height, and both ends are polished to ensure that there are no uneven ends of the composite tube.
(6) Foam filling: The foam material components are mixed in equal proportions and then poured into the bamboo composite tube. The two ends of the tube are compacted by heavy objects and left for 10 min for natural foaming. When the volume no longer changes, the heavy objects are removed, and the specimen is finished.

### 3.2. Specimen Preparation

A total of 36 bamboo composite tubes with a length-to-diameter ratio (R) of 2 were prepared by using two types of tube inner diameters (D), three types of tube wall winding layers (L) and polyurethane foam filling as the parameters that changed, with a total of 12 different groups of bamboo composite tubes, each with 3 identical specimens, to eliminate random influences. The fiber orientation was parallel to the loading direction. The specific parameters of each specimen are shown in Table 3. The naming rules of the specimens are as follows: taking D100L15R2F as an example, the inner diameter is 100 mm, the number of winding layers is 15, the length–diameter ratio is 2.0, and the bamboo composite tube is filled with polyurethane foam.

### 3.3. Mechanical Testing Setup and Procedure

**Figure 3:**
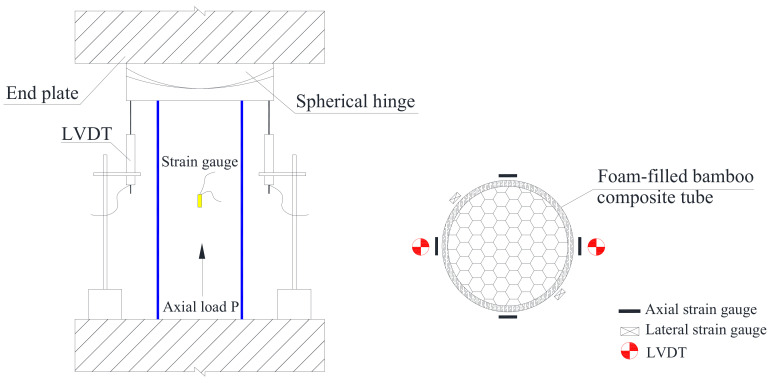
Layout of the test equipment and strain gauge.

Axial compression tests were carried out on a 3000 kN high-stiffness compression testing machine (YAW-G3000). Vertical linear variable displacement transducers (LVDTs) were used to measure the axial compression deformation. To monitor the development of the longitudinal strain in the specimens, strain gauges were attached to the surface of the middle longitudinal section of the specimens. Strain and displacement data acquisition was carried out synchronously by TDS-530, and the acquisition frequency was unified to 3 s. At the beginning of each test, 10 kN was preloaded three times to eliminate the gap between the testing machine and the specimens and to ensure that the LVDT and strain gauge worked normally. The loading control mode was displacement control, the uniaxial axial compression loading rate was 0.3 mm/min, and the loading rate was adjusted to 1.0 mm/min after the failure of the specimen. The specimen device and strain gauge arrangement are shown in Figure 3.

## 4. Results and Discussion

### 4.1. Failure Modes

**Figure 4:**
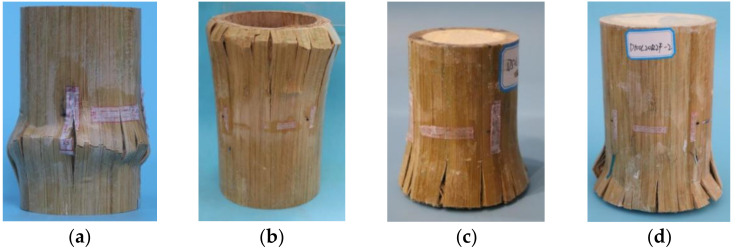
Failure modes of polyurethane foam-filled bamboo composite tubes. (**a**) D50 buckling; (**b**) D100 buckling; (**c**) D50F bearing; (**d**) D100F bearing.

As shown in Figure 4, the failure of the unfilled bamboo composite tube started with local buckling at the end of the tube. With the further increase in load, the buckling phenomenon became increasingly prominent. Several fine longitudinal cracks developed and expanded axially in the bulging part and were distributed in the circumferential direction. Finally, multiple obvious cracks were formed, and the bearing capacity was lost, which was characterized by bending and splitting between layers. The failure mode of the unfilled bamboo composite tube was buckling failure. The cracks developed gently and expanded progressively, whose depth was shallow and length was short. The bearing capacity was not completely lost, and the crack propagation to failure took a long time, which indicated ductile failure. The failure of polyurethane foam-filled bamboo composite tubes began with the formation of fine cracks at the ends of the tubes. The form and distribution of cracks were similar to those of unfilled tubes. However, the failure of the foam-filled bamboo composite tube was not as obvious as that of the unfilled bamboo composite tube. The bearing capacity was suddenly lost, and the end was crumpled and bifurcated. After reaching the bearing limit of bamboo fiber, the fracture of the fiber led to damage. The failure of the polyurethane foam-filled bamboo composite tube was bearing failure and was considered brittle failure.

Due to the lack of internal and external constraints, the buckling of bamboo fiber was bidirectional, and the bamboo fiber layer on the inner side buckled to the inside, while the bamboo fiber layer on the outside was the opposite. The presence of polyurethane foam filler enhanced the lateral support of bamboo composite tubes. Under biaxial stress, bamboo fibers uniformly deformed to the outside of the tube wall, and the trend of internal buckling of inner bamboo fibers was restrained. The entire thickness of the bamboo fiber layer worked together. Therefore, the buckling phenomenon of the foam-filled tube was not significant, resulting in the different failure modes of the two tubes.

### 4.2. Load–Displacement Curves

**Figure 5:**
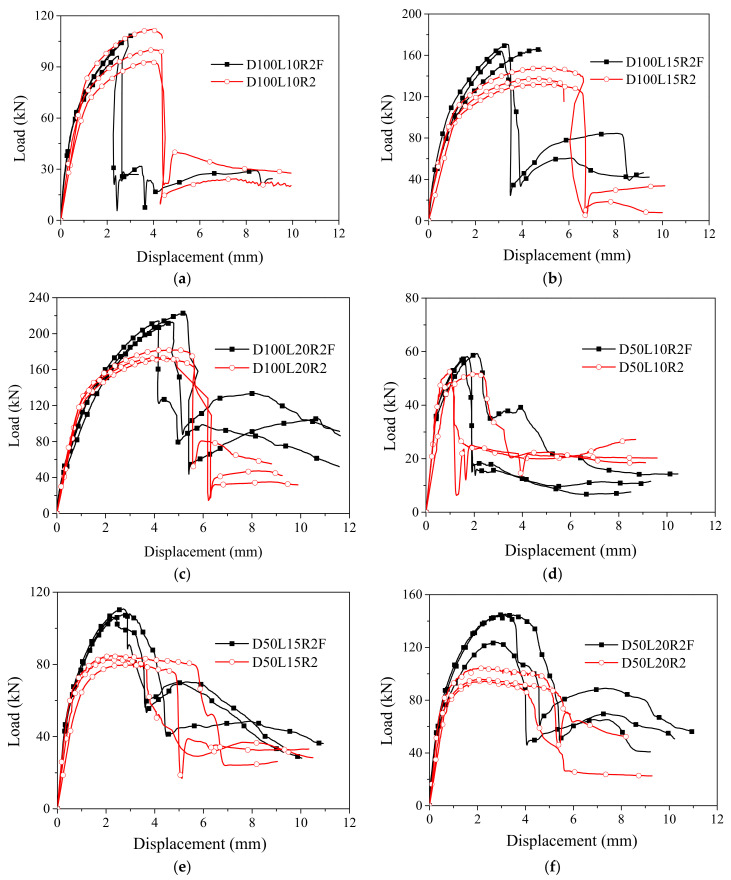
Comparison of load–displacement curves between foam-filled and unfilled bamboo composite tubes. (**a**) D100L10R2F; (**b**) D100L15R2F; (**c**) D100L20R2F; (**d**) D50L10R2F; (**e**) D50L15R2F; (**f**) D50L20R2F.

The load–displacement comparison curves of unfilled and polyurethane foam-filled bamboo composite tubes are shown in Figure 5. The load–displacement curves can be divided into three stages: linear elastic growth, elastoplastic strengthening and destruction. In the linear elastic growth stage, the load increases rapidly without any phenomena. After that, it enters the stage of elastic-plastic strengthening, and microcracks with short lengths and shallow depths gradually form. With the development of cracks, the load gradually reaches the peak value, and the bamboo fiber is broken. The load jumps to the stage of low-level load, and the curve enters a new stable state.

The effect of polyurethane foam filler on increasing the ultimate load of the bamboo composite tube is remarkable. The average ultimate load of the foam-filled tube was increased by 2.5–40.8%, and with the increase in the winding layer number, the beneficial effect of the foam filler on the ultimate load increased gradually. However, the effect of the bamboo composite tube with a thickness of 5 mm (L = 10) was not obvious. This is because the ultimate displacement of the polyurethane foam-filled bamboo composite tube with a thickness of 5 mm (L = 10) was approximately 2 mm and was much smaller than that of the foam-filled tube with a thickness of 7.5 mm (L = 15) and a wall thickness of 10 mm (L = 20). This was also observed in the unfilled bamboo composite tube. According to the static compression test of polyurethane foam, polyurethane foam basically failed to function under a deformation of 2 mm, and thus, the effect on the ultimate load was very small.

The highest load point is defined as the peak point, and the sharp drop point is defined as the ultimate point. The polyurethane foam-filled bamboo composite tube was damaged immediately after reaching the ultimate load. It did not have the deformation capacity after the maximum carrying capacity when the load capacity was not significantly reduced; that is, the peak point coincided with the ultimate point. In Figure 5, the comparison shows that the addition of polyurethane foam filler had a negative effect on the ultimate displacement. Compared with the corresponding unfilled bamboo composite tube, the ultimate displacement of the bamboo composite tube decreased to 11.0%-42.4% after foam filling. The addition of foam filler weakened the deformation capacity of the bamboo composite tube under the action of bidirectional stress, and the inward deformation of the bamboo fiber was restrained. Foam-filled specimens still maintained a certain load level without completely losing their bearing capacity, which is similar to that of unfilled bamboo composite tubes. The difference is that the platform loading of the foam-filled specimens was generally higher than that of the corresponding unfilled specimens, especially in the tube specimens with wall thicknesses of 7.5 mm (L = 15) and 10 mm (L = 20). The polyurethane foam filler contributes to the higher platform loading at the later stage, which may be beneficial for enhancing the energy absorption function of the bamboo composite tube.

### 4.3. Stress–Strain Curves

**Figure 6:**
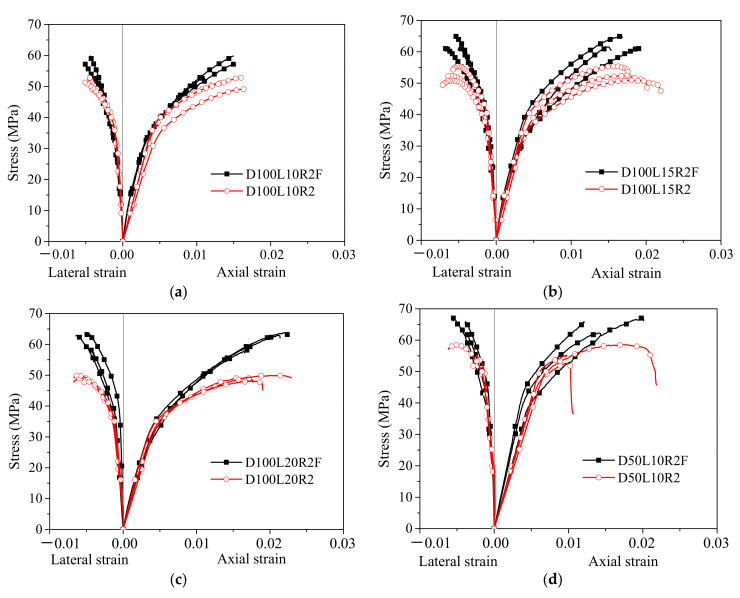

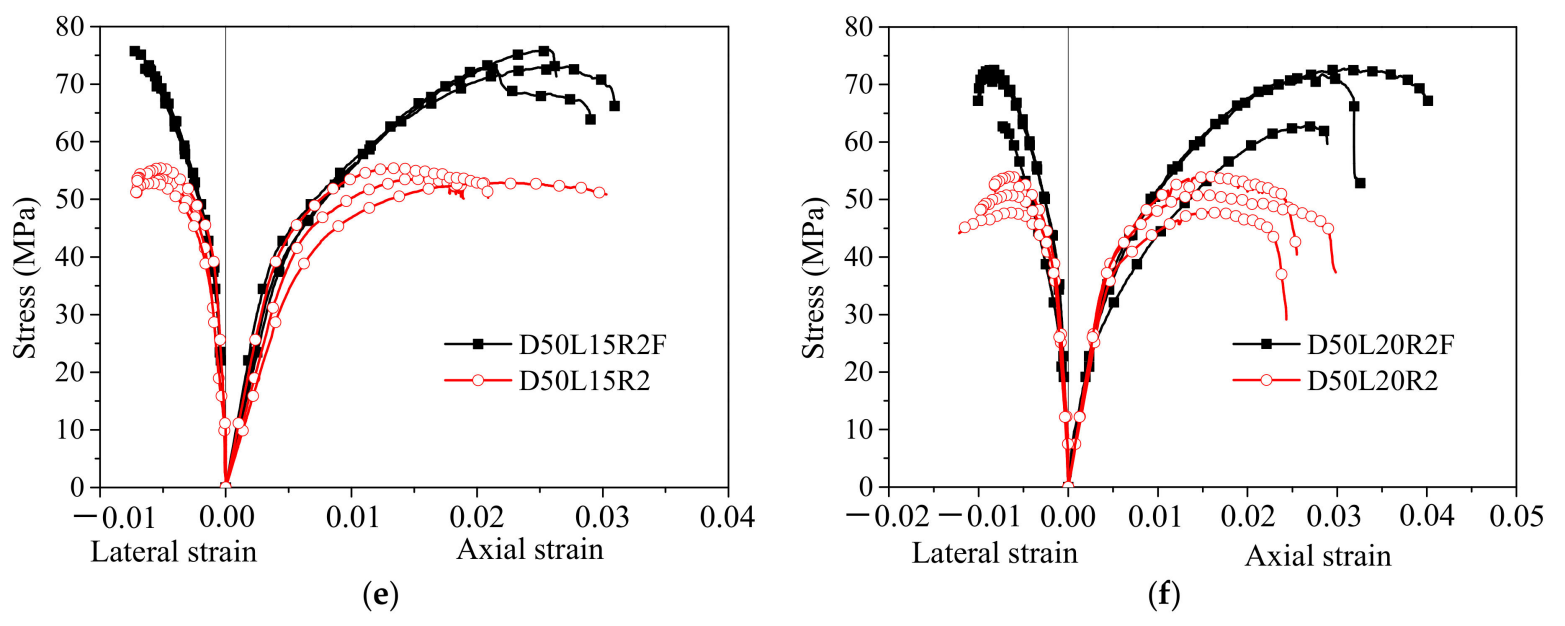
Comparison of stress–strain curves between foam-filled and unfilled bamboo composite tubes. (**a**) D100L10R2F; (**b**) D100L15R2F; (**c**) D100L20R2F; (**d**) D50L10R2€(**e**) D50L15R2F; (**f**) D50L20R2F.

**Figure 7:**
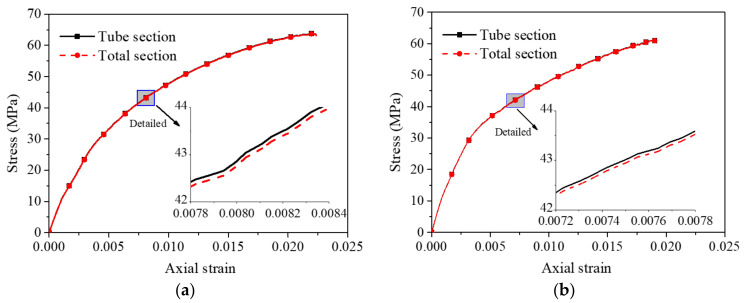
Comparison of the axial stress considering different compression areas. (**a**) D100L20R2F-1; (**b**) D100L15R2F-1.

A comparison of the stress–strain curves of unfilled bamboo and polyurethane foam-filled bamboo composite tubes is shown in Figure 6. The axial strain is positive, and the lateral strain is negative in the figure. The ratio between the load recorded by the test and the actual cross-sectional area of the bamboo composite tube, which is the axial stress, was obtained without considering the cross-sectional area of the polyurethane foam. The experimental results show that the polyurethane foam was still in the linear elastic stage when the foam-filled bamboo composite tube reached the ultimate bearing state. At this stage, the maximum contribution of the axial stress of polyurethane foam was no more than 0.1 MPa. Taking the test results of D100L20R2F-1 and D100L15R2F-1 as examples in Figure 7, regardless of whether the full cross-sectional area or the cross-sectional area of the bamboo composite tube was taken into account in the calculation of axial stress, the error was only 0.07 MPa, accounting for only 0.11%. Therefore, it is reasonable to take the ratio of the load and the actual section of the bamboo composite tube as the axial stress of the specimens.

In the stress–strain curves in Figure 6, the unfilled and foam-filled bamboo composite tubes show the same elastic-plastic characteristics. The stress–strain curves showed linear growth at the beginning, which is the elastic stage. With the increase in strain, the stress grew slowly, and this stage is the plastic stage. Polyurethane foam filler did not change the elastic modulus of the bamboo composite tube. Compared with the average modulus of 9.0 GPa of the unfilled bamboo composite tube, the 0.6 MPa modulus of the polyurethane foam was very small. The elastic modulus of the foam-filled bamboo composite tube was stable in the range of 7.3–10.5 GPa, and the average elastic modulus was 9.1 GPa. Table 4 shows the results of the bamboo composite tube under axial compression. The average peak stress of polyurethane foam-filled bamboo composite tubes was 56.84–74.06 MPa, corresponding to that of the unfilled bamboo composite tube increasing by 11.0–37.1%, showing a gradual increase in the magnitude of stress enhancement along with an increase in the winding layer number. Polyurethane foam filler greatly enhanced the strengthening effect of the bamboo composite tube in the plastic section, and the peak stress markedly increased, especially in the 15-winding-layer and 20-winding-layer foam-filled bamboo-wound composites. Since the maximum displacement of the 10-winding-layer foam-filled bamboo composite tube is much lower than that of the tube, the polyurethane foam has a better effect. The average peak strain of the foam-filled composite tube was 0.0137–0.0290, and the peak strain was greatly improved, especially in the D50F group, in which the peak strain increased by 23.4%, 49.1% and 83.3% when increasing from 10 to 20 layers, respectively. The growth of the winding layer number is beneficial for increasing the peak strain of the foam-filled bamboo composite tube. The increase in tube diameter will cause a decrease in peak strain, which is notable in D100F and D50F. The peak strain of the former was lower than that of the latter. The drop was up to 10.5%, 49.1% and 45.0%, respectively.

The load-carrying capacity of low-density polyurethane foam is negligible. The bamboo composite tube is the main contributor to the load-carrying capacity of the foam-filled bamboo composite tube, and it also plays a role in restraining the lateral deformation of the inner polyurethane foam and its recovery. Polyurethane foam significantly improved the structural stability of the bamboo composite tube wall. The combination of the two eventually led to a foam-filled bamboo composite tube with a higher loading capacity. The combination effect highlighted the “1 + 1 > 2” combination concept, which confirmed the rationality and feasibility of the combination of bamboo composite tubes and polyurethane foam.

### 4.4. Energy Absorption Characteristics

To evaluate the energy absorption characteristics of polyurethane foam-filled bamboo composite tubes, in addition to the ultimate load *P_u_*, another four commonly used energy absorption evaluation indices were introduced.

(1) The absorbed energy *AE* is the area under the load–displacement response curve:

(1) $$AE=\int_{0}^{l}Pdl$$

(2) The specific absorbed energy *SAE* is the absorbed energy per unit mass of the specimen:

(2) $$SAE=\frac{AE}{m}$$

(3) The average crush load *P_avg_* is the average load compressed to a certain displacement:

(3) $$P_{avg}=\frac{AE}{l}$$

(4) The crush force efficiency *CFE* is the ratio of the average crush load to the ultimate load:

(4) $$CFE=\frac{P_{avg}}{P_{\max}}$$

where *P* is the compressive load of the tube at a certain time; *l* is the displacement corresponding to the load at that moment; and *m* is the mass of the specimen.

Table 5 shows *P_u_*, *AE*, *SAE*, *P_avg_* and *CFE* when the compression displacement is 8 mm. For the tube with an ultimate displacement of less than 8 mm, the parameters were calculated for the actual displacement. It can be seen in Table 5 that all energy absorption indices increased with increasing tube wall thickness, regardless of whether the tube was unfilled or foam-filled. Compared with the process of increasing the wall thickness from 7.5 mm to 10 mm, each energy absorption index had a greater proportion of improvement with the increase in the wall thickness from 5 mm to 7.5 mm. The increase in the wall thickness from 5 mm to 7.5 mm had more notable effects on enhancing energy absorption. For example, from D50L10R2 to D50L15R2, *SAE*, *P_avg_* and *CFE* increased by 80.1%, 198.6% and 61.2%, respectively, while from D50L15R2 to D50L20R2, the corresponding indices only increased by 7.6%, 33.3% and 4.3%, respectively. The influence of the tube diameter on the energy absorption performance of bamboo composite tubes is more complex. The *SAE* greatly decreased by more than 45% with increasing tube diameter, while *P_avg_* increased with increasing tube diameter. The *SAE* of the foam-filled tube was higher than that of the unfilled tube, and the increase in *P_avg_* was lower than that of the unfilled tube. In terms of *CFE*, that of D100F was lower than that of D50F, while that of D100 slightly increased compared with D50, but the overall increase was not large. From the point of view of the *AE*, increases in wall thickness and tube diameter had obvious beneficial effects on the *AE*.

As indicated by the comparison of the load–displacement curves of unfilled and foam-filled bamboo composite tubes in Figure 5, the general trend of load–displacement curves of foam-filled tubes was similar to that of the unfilled tubes, showing a progressive and stable failure mode. The lateral expansion of foam produces pressure on the inner surface of the tube, which enhances the friction between the foam and the bamboo tube and makes the two objects resist the compressive load at the same time, thus improving the ultimate load of the bamboo composite tube. The ultimate displacement of the foam-filled tubes was smaller than that of the corresponding unfilled tubes. However, the polyurethane foam filler can contribute to a higher platform load. These two aspects have advantages and disadvantages for the energy absorption of bamboo composite tubes. The influence of polyurethane foam on the energy absorption characteristics of the bamboo composite tube needs to be comprehensively considered.

**Figure 8:**
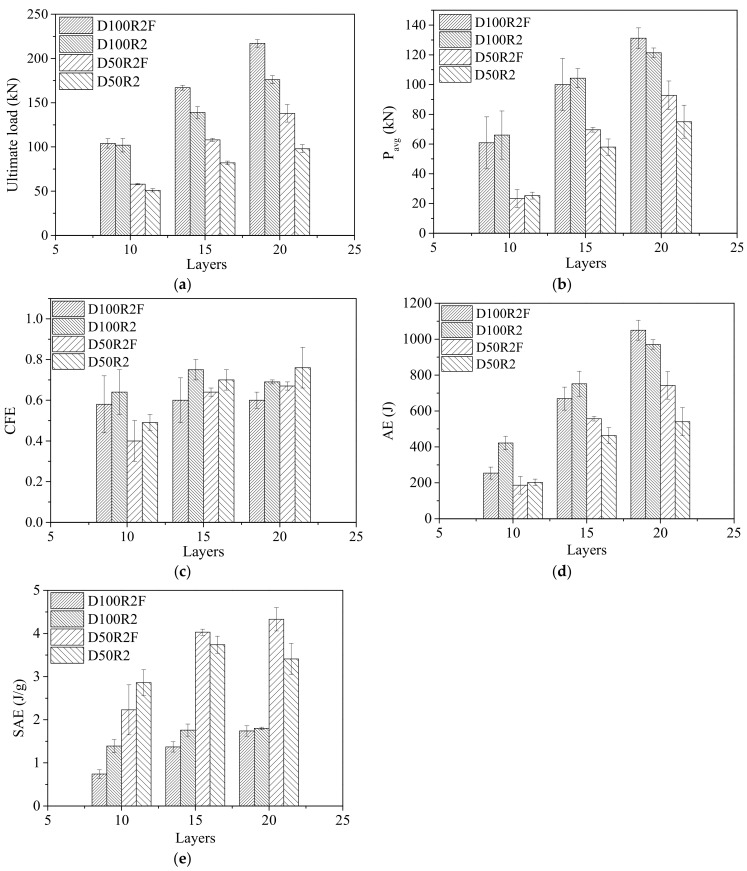
Effect of foam filler on evaluation indices of the energy absorption of bamboo composite tubes. (**a**) Ultimate load; (**b**) average crush load; (**c**) crush force efficiency; (**d**) absorbed energy; (**e**) specific absorbed energy.

The effects of foam filler on the *P_u_*, *AE*, *SAE*, *P_avg_* and *CFE* of bamboo composite tubes are shown in Figure 8. As shown in Figure 8a, the ultimate loads of all foam-filled bamboo composite tubes were greater than those of the corresponding unfilled tubes. It is shown that the use of foam filler is beneficial to the ultimate load of bamboo composite tubes. Compared with the tubes with 10 layers, the foam filler had a more significant effect on the ultimate load for bamboo composite tubes with 15 and 20 layers. As shown in Figure 8b, the average crush load of the foam-filled tubes was not significantly different from that of the unfilled tube. Since it is the statistical data of compressive displacement to 8 mm, the change rule of absorbed energy is consistent with that of the average crush load, as shown in Figure 8b,d. For the tube with a diameter of 100 mm, the absorbed energies of the foam-filled tubes were 0.68, 0.88 and 1.08 times those of the corresponding unfilled tubes, respectively. For tubes with diameters of 50 mm, their values were 0.92, 1.20 and 1.32 times those of the corresponding unfilled tubes. The energy absorption of the polyurethane foam filler can be neglected. In terms of energy absorption, the combination effect of the foam filler and the bamboo composite tube is not excellent, which shows that the addition of the foam filler does not significantly enhance the energy absorption of the bamboo composite tube, but on the contrary, it will lead to a certain reverse effect. This effect is mainly due to the reduction in the ultimate displacement, and the foam filler does not enhance the load holding length of the bamboo composite tubes; therefore, the absorbed energy decreases instead of rising. The main reason why the load cannot be maintained is that the foam filler inhibits the inward shrinkage deformation of bamboo composite tubes, leading to the splitting failure of composite tubes after reaching the ultimate load, and the load drops rapidly. In particular, the composite tubes with fewer winding layers are more prone to splitting failure, and the foam filler has a greater negative impact on its energy absorption. Thus, the absorbed energies of most L10F and L15F specimens were smaller than those of specimens L10 and L15. As shown in Figure 8c, with the existence of foam filler, the crush force efficiency was markedly reduced because the use of foam filler increased the ultimate load, but the average crush load had no obvious enhancement trend; in contrast, there was a reduction, resulting in a smaller crush force efficiency ratio. In Figure 8e, it can be seen that the specific absorbed energy of the tubes decreases with increasing tube length. Therefore, reducing the height of the bamboo composite tubes and increasing the number of winding layers of the bamboo composite tubes can effectively increase the positive effect of the foam filler on energy absorption.

## 5. Conclusions

In this paper, axial compression tests on different types of polyurethane foam-filled bamboo composite tubes were carried out. The failure modes, load–displacement responses and stress–strain relationships of the composite tubes with the corresponding unfilled tubes were compared. The effects of polyurethane foam filler on the failure modes and mechanical properties of the bamboo winding composite tubes were analyzed. The following conclusions can be drawn:

(1) The failure of polyurethane foam-filled bamboo composite tubes began with the formation of fine cracks at the ends of the tubes, and finally, the bamboo fibers were crumpled and bifurcated. There were multiple longitudinal cracks in the circumferential distribution. The failure, termed bearing failure, occurred suddenly and was classified as brittle failure. The polyurethane foam filler greatly enhanced the plastic section strengthening effect of the bamboo composite tube, and the axial peak stress was markedly improved, but it did not change the initial stiffness of the tube.
(2) The energy absorption characteristics of unfilled and foam-filled bamboo composite tubes were analyzed with the following five evaluation indices: ultimate load, absorbed energy, specific absorbed energy, average crush load and crush force efficiency. Reducing the height of the bamboo composite tubes and increasing the number of winding layers of the bamboo composite tubes can effectively increase the positive effect of the foam filler on energy absorption.
(3) The loading capacity of the polyurethane foam-filled bamboo composite tube was significantly higher than that of the corresponding unfilled bamboo composite tube. The bamboo composite tube was the main contributor to the load-carrying capacity of the foam-filled bamboo composite tube, and it also played a role in restraining the lateral deformation of the inner polyurethane foam and restraining its recovery. The polyurethane foam significantly improved the structural stability of the bamboo composite tube. Both of them had good complementarity in performance. The combination effect highlights the concept of the “1 + 1 > 2” combination. This research fully confirms the rationality and feasibility of the combination of bamboo composite tubes and polyurethane foam.

## Figures and Tables

**Table 1:** Mechanical properties of bamboo slices.

Group	Mechanical Indices	Average	Coefficient of Variation	Failure Mode
t = 5 mm (Compression)	Ultimate strength (MPa)	55.40	5.40%	1S9L
	Elastic limit stress (MPa)	38.47	10.81%	
t = 7.5 mm (Compression)	Ultimate strength (MPa)	59.73	4.03%	2S8L
	Elastic limit stress (MPa)	39.33	9.89%	
t = 10 mm (Compression)	Ultimate strength (MPa)	54.29	7.98%	4S6L
	Elastic limit stress (MPa)	40.40	13.00%	
t = 0.5 mm (Tension)	Ultimate strength (MPa)	95.03	5.10%	Serrated failure
	Ultimate strain	0.0077	3.74%	
	Modulus of elasticity (GPa)	12.12	6.79%	

Note: xSyL indicates that x specimens show splitting failure, and y specimens show buckling failure.

**Table 2:** Results of the static compression test for polyurethane foam.

Specimen	Load *F*_pu_ (N)	Density *ρ* (kg·m^−3^)	Stress *σ*_10_ (kPa)	Nominal Modulus of Elasticity *E* (kPa)
PU-1	3230	29.94	51	526
PU-2	2928	31.96	55	588
PU-3	3144	31.70	59	663
PU-4	3218	30.66	67	861
PU-5	3678	33.24	71	842
Average	3240	31.50	60	696
Variation	8.44%	4.02%	13.38%	21.61%

Notes: The load *F*_pu_ is the corresponding load value when the displacement reached 42.5 mm, and the compressive stress *σ*_10_ is the stress value corresponding to 10% relative deformation.

**Table 3:** Design parameters of the bamboo composite tube.

Group	Specimen	Length *H* (mm)	Diameter *D* (mm)	Winding layers *L*	Wall Thickness *t* (mm)	Filler
D50	D50L10R2-1/2/3	100	50	10	5	N/A
	D50L15R2-1/2/3	100	50	15	7.5	N/A
	D50L20R2-1/2/3	100	50	20	10	N/A
D100	D100L10R2-1/2/3	200	100	10	5	N/A
	D100L15R2-1/2/3	200	100	15	7.5	N/A
	D100L20R2-1/2/3	200	100	20	10	N/A
D50F	D50L10R2F-1/2/3	100	50	10	5	Foam
	D50L15R2F-1/2/3	100	50	15	7.5	Foam
	D50L20R2F-1/2/3	100	50	20	10	Foam
D100F	D100L10R2F-1/2/3	200	100	10	5	Foam
	D100L15R2F-1/2/3	200	100	15	7.5	Foam
	D100L20R2F-1/2/3	200	100	20	10	Foam

**Table 4:** The results of the bamboo composite tubes under axial compression.

Group	Specimen	Ultimate Load *P*_max_ (kN)	Peak Stress *σ*_p_ (MPa)	Peak Strain *ε*_p_	Ultimate Stress *σ*_cu_ (MPa)	Ultimate Strain *ε*_cu_	Modulus of Elasticity *E* (MPa)
D50	D50L10R2-1	53.0	55.27	0.0097	55.27	0.0097	8300
	D50L10R2-2	48.6	52.78	0.0102	51.76	0.0103	8000
	D50L10R2-3	51.8	58.68	0.0172	57.70	0.0200	7400
	D50L15R2-1	82.7	53.66	0.0156	50.14	0.0189	8300
	D50L15R2-2	79.8	52.93	0.0219	49.61	0.0325	7300
	D50L15R2-3	84.8	55.48	0.0131	50.28	0.0209	10,800
	D50L20R2-1	94.4	47.84	0.0166	43.66	0.0229	8500
	D50L20R2-2	104.5	54.17	0.0166	50.99	0.0237	9400
	D50L20R2-3	95.8	50.82	0.0150	46.12	0.0283	9600
D100	D100L10R2-1	93.1	49.19	0.0164	48.55	0.0164	7800
	D100L10R2-2	100.3	52.99	0.0158	52.46	0.0161	9300
	D100L10R2-3	111.9	51.45	0.0140	50.38	0.0146	9400
	D100L15R2-1	137.6	52.38	0.0168	51.27	0.0193	9400
	D100L15R2-2	132.0	50.95	0.0175	47.79	0.0219	9700
	D100L15R2-3	147.7	55.46	0.0160	52.44	0.0175	10,000
	D100L20R2-1	171.8	49.18	0.0159	47.16	0.0176	7700
	D100L20R2-2	182.2	49.99	0.0201	49.20	0.0229	8400
	D100L20R2-3	173.6	48.13	0.0179	47.35	0.0189	9300
D50F	D50L10R2F-1	58.3	66.04	0.0121	66.04	0.0121	11,400
	D50L10R2F-2	59.4	67.28	0.0201	67.28	0.0201	8500
	D50L10R2F-3	57.4	62.34	0.0138	62.34	0.0138	10,500
	D50L15R2F-1	106.5	73.06	0.0215	73.06	0.0215	9300
	D50L15R2F-2	108.1	73.12	0.0282	73.12	0.0282	12,300
	D50L15R2F-3	110.8	76.01	0.0258	76.01	0.0258	10,100
	D50L20R2F-1	145.1	71.90	0.0288	71.05	0.0297	8000
	D50L20R2F-2	124.3	62.99	0.0266	61.90	0.0285	7900
	D50L20R2F-3	145.5	72.91	0.0315	67.47	0.0400	8000
D100F	D100L10R2F-1	107.1	59.90	0.0149	59.90	0.0149	8800
	D100L10R2F-2	96.8	53.10	0.0109	53.10	0.0109	9300
	D100L10R2F-3	108.9	57.53	0.0151	57.53	0.0151	9300
	D100L15R2F-1	165.9	61.12	0.0191	61.12	0.0191	8600
	D100L15R2F-2	164.0	61.58	0.0149	61.58	0.0149	8500
	D100L15R2F-3	170.7	64.98	0.0166	64.98	0.0166	10,100
	D100L20R2F-1	223.0	63.83	0.0223	63.83	0.0223	6500
	D100L20R2F-2	214.7	58.29	0.0167	58.29	0.0167	6900
	D100L20R2F-3	213.1	63.04	0.0209	63.04	0.0209	8600

**Table 5:** Energy absorption indices of unfilled and foam-filled bamboo composite tubes.

Group	Specimen	Mass *m* (g)	Wall Thickness *t* (mm)	Ultimate Load *P*_max_ (kN)	Absorbed Energy *AE* (J)	Specific Absorbed Energy *SAE* (J/g)	Average Crush Load *P*_avg_ (kN)	Crush Force Efficiency *CFE*
D100F	D100L10R2F-1	342.3	5.4	107.1	219.7	0.64	65.68	0.61
	D100L10R2F-2	341.9	5.5	96.8	299.1	0.87	37.39	0.39
	D100L10R2F-3	345.3	5.7	108.9	243.5	0.71	79.42	0.73
	D100L15R2F-1	463.1	8.0	165.9	597.2	1.29	123.87	0.75
	D100L15R2F-2	513.2	7.9	164	656.4	1.28	82.05	0.50
	D100L15R2F-3	485.0	7.8	170.7	753.1	1.55	94.14	0.55
	D100L20R2F-1	601.3	10.1	223	1041.6	1.73	130.20	0.58
	D100L20R2F-2	615.7	10.6	214.7	986.2	1.60	123.28	0.57
	D100L20R2F-3	589.7	9.8	213.1	1121.3	1.90	140.16	0.66
D100	D100L10R2-1	287.2	5.7	93.1	403.8	1.41	50.48	0.54
	D100L10R2-2	303.3	5.7	100.3	474.1	1.56	59.26	0.59
	D100L10R2-3	322.3	6.5	111.9	387.6	1.20	88.43	0.79
	D100L15R2-1	418.0	7.8	137.6	654.1	1.56	112.98	0.82
	D100L15R2-2	423.2	7.7	132.0	783.1	1.85	97.89	0.74
	D100L15R2-3	434.4	7.9	147.7	817	1.88	102.13	0.69
	D100L20R2-1	527.7	10.1	171.8	961.9	1.82	120.24	0.70
	D100L20R2-2	556.2	10.5	182.2	1006.6	1.81	125.83	0.69
	D100L20R2-3	536.4	10.4	173.6	944.1	1.76	118.01	0.68
D50F	D50L10R2F-1	84.1	5.1	58.3	156.5	1.86	19.56	0.34
	D50L10R2F-2	83.8	5.1	59.4	255.5	3.05	31.94	0.54
	D50L10R2F-3	82.3	5.3	57.4	147.7	1.79	18.46	0.32
	D50L15R2F-1	139.4	8.0	106.5	561.7	4.03	70.21	0.66
	D50L15R2F-2	137.0	8.1	108.1	539.5	3.94	67.44	0.62
	D50L15R2F-3	138.7	8.0	110.8	570	4.11	71.25	0.64
	D50L20R2F-1	178.4	10.6	145.1	794.6	4.45	99.33	0.68
	D50L20R2F-2	160.6	10.4	124.3	634.1	3.95	79.26	0.64
	D50L20R2F-3	174.2	10.5	145.5	799.5	4.59	99.94	0.69
D50	D50L10R2-1	74.2	5.5	53.0	196.6	2.65	24.58	0.46
	D50L10R2-2	69.8	5.3	48.6	184.1	2.64	23.01	0.47
	D50L10R2-3	69.2	5.1	51.8	227.1	3.28	28.39	0.55
	D50L15R2-1	121.3	8.4	82.7	422.4	3.48	52.80	0.64
	D50L15R2-2	117.4	8.2	79.8	443.9	3.78	55.49	0.70
	D50L15R2-3	132.0	8.3	84.8	524.4	3.97	65.55	0.77
	D50L20R2-1	151.8	10.4	94.4	474.9	3.13	59.36	0.63
	D50L20R2-2	165.9	10.2	104.5	650.9	3.92	81.36	0.78
	D50L20R2-3	155.7	10.0	95.8	495.9	3.18	84.37	0.88

Notes: For D100L10R2F-1, D100L10R2F-3, D100L15R2F-1, D100L10R2-3, D100L15R2-1 and D50L20R2-3, the displacement is taken as the actual value.

## Data Availability

The data presented in this study are available on request from the corresponding author.
